# 3D Breast Tumor Models for Radiobiology Applications

**DOI:** 10.3390/cancers13225714

**Published:** 2021-11-15

**Authors:** Akhilandeshwari Ravichandran, Julien Clegg, Mark N. Adams, Madison Hampson, Andrew Fielding, Laura J. Bray

**Affiliations:** 1 School of Mechanical, Medical and Process Engineering, Queensland University of Technology (QUT), Brisbane, QLD 4000, Australia; akhilandeshwari.ravichandran@qut.edu.au (A.R.); julien.clegg@hdr.qut.edu.au (J.C.); madison.hampson@connect.qut.edu.au (M.H.); 2 ARC Training Centre for Cell and Tissue Engineering Technologies, Queensland University of Technology (QUT), Brisbane, QLD 4000, Australia; mn.adams@qut.edu.au; 3 School of Biomedical Sciences, Queensland University of Technology, Brisbane, QLD 4000, Australia; 4 School of Chemistry and Physics, Queensland University of Technology, Brisbane, QLD 4000, Australia; a.fielding@qut.edu.au

**Keywords:** 3D radiobiology, in vitro breast cancer models, radiation therapy, radiosensitizers

## Abstract

**Simple Summary:**

Breast cancer is one of the most commonly diagnosed cancers worldwide and remains a leading cause of cancer-associated death in women. Radiation therapy is frequently used and plays a key role in the clinical treatment of breast cancers. A better understanding of the biological mechanisms that contribute to the response of cell and tissues to radiation therapy will allow for more targeted and personalized treatment plans in the future. This review investigates the use of three-dimensional (3D) models for the study of radiation therapy in the context of breast cancer to help inform future directions for the field.

**Abstract:**

Breast cancer is a leading cause of cancer-associated death in women. The clinical management of breast cancers is normally carried out using a combination of chemotherapy, surgery and radiation therapy. The majority of research investigating breast cancer therapy until now has mainly utilized two-dimensional (2D) in vitro cultures or murine models of disease. However, there has been significant uptake of three-dimensional (3D) in vitro models by cancer researchers over the past decade, highlighting a complimentary model for studies of radiotherapy, especially in conjunction with chemotherapy. In this review, we underline the effects of radiation therapy on normal and malignant breast cells and tissues, and explore the emerging opportunities that pre-clinical 3D models offer in improving our understanding of this treatment modality.

## 1. Introduction

Breast cancer is a leading cause of cancer-associated death in women [1]. Breast cancers have been categorized into three major subtypes based on the presence of the hormonal receptors-estrogen receptor (ER), progesterone receptor (PR), and human epidermal growth factor 2 (HER2). Breast cancers are also classified into Stages 0, I, II, III and IV depending on the severity of the cancer, ranging from least invasive to highly metastatic cancers respectively. Clinical management of breast cancer is carried out using a combination of therapeutic modalities including chemotherapy, surgery, radiation therapy and palliative care. In the case of non-metastatic breast cancer, almost 50% of patients are treated by tumor resection through a localized lumpectomy followed by radiation therapy [2]. More than half of patients with stage III breast cancers are treated by a mastectomy [2]. This is usually followed up with systemic hormonal therapy, chemotherapy, or breast tissue irradiation. Once the cancer has metastasized to distant organs in Stage IV, the predominant treatment modalities have been radiation therapy and/or chemotherapy, used in over 56% of patients [2].

Evidently, radiation therapy is a consistent modality used in the clinical intervention of breast cancers [2]. In this review, we briefly discuss the biological effects of radiation therapy followed by the current preclinical tools available to study these effects for breast cancers. Several preclinical, in vitro irradiation studies have been conducted using two-dimensional (2D) cancer cell cultures [3]. Recently, there has been increased appreciation for using three-dimensional (3D) tissue models including spheroid cultures (scaffold-free) or tissue cultures with scaffolds and hydrogels to study the effects of irradiation [3]. The results observed in these models are increasingly reliable as these structures mimic the native tissue organization and concomitant cellular characteristics. Particularly, this review focuses on the use of 3D breast cancer models for the study of radiation therapy.

## 2. Radiation Induced Cell-Death

Radiation therapy or radiotherapy (RT) typically uses ionizing radiation to inhibit the uncontrolled growth of cancer cells. RT is typically fractionated i.e., multiple low dose fractions are delivered resulting in a cumulative high dose, with the aim of minimizing normal cell toxicity and tissue damage [4,5]. RT induces a myriad of physiological effects upon the body and modulates changes in cell behavior. The biological effects of RT, caused by the direct or indirect effects upon cells, are characterized by the hallmark effect of DNA damage. This DNA damage inhibits cellular reproduction, reduces cellular metabolism and induces apoptotic signaling pathways [6]. In fact, in the case of low linear energy transfer (LET) x-rays and gamma rays, up to 60% of the cellular damage that is observed is due to the indirect effects. This places the indirect mechanisms of action as a possible avenue for further research [7]. With the complementary effects of both direct and indirect RT mechanisms of action, tumors and cancers will typically respond in a positive way to treatments.

### 2.1. Direct Effects

Within the direct action, the ionizing radiation hits DNA molecules directly within the nuclei of targeted cells. This disrupts the DNA structure resulting in double-strand breaks (DSBs) or single-strand breaks (SSBs) in the DNA [6]. DSBs are the most cytotoxic genomic lesion as failure to repair these damages results in genomic instability or cell death [8]. Two pathways exist to repair DSBs; homologous recombination (HR) and non-homologous end joining (NHEJ). HR is restricted to the S and G2 cell cycle phases when a sister chromatid is available as a homologous template [8,9]. HR is a highly complex and coordinated pathway, which is broadly initiated by the MRE11-RAD50-NSB1 (MRN) complex, which, in conjunction with exonucleases, resect the DSBs. This processing exposes single-strand DNA which is rapidly coated by replication protein A (RPA) [10] followed by recruitment of the BRCA1 protein. The BRCA1 and 2 proteins promote the Rad51-mediated search for, and invasion of, the homologous template to form a Holliday junction and continued downstream DNA synthesis and dissolution of the junction

Unlike HR, NHEJ is active throughout all phases of the cell cycle and functions without a homologous template and directly ligating the DNA ends [8]. This pathway is initiated by the Ku heterodimer, followed by recruitment and activation of the DNA-dependent protein kinase (DNA-PKcs) [11]. This active complex bridges the DSB to enable either DSB end digestion or gap-filling via recruitment of several enzymes (reviewed in [12]) prior to DNA end ligation by the DNA Ligase IV/XRCC4 complex [8,12].

**Figure 1:**
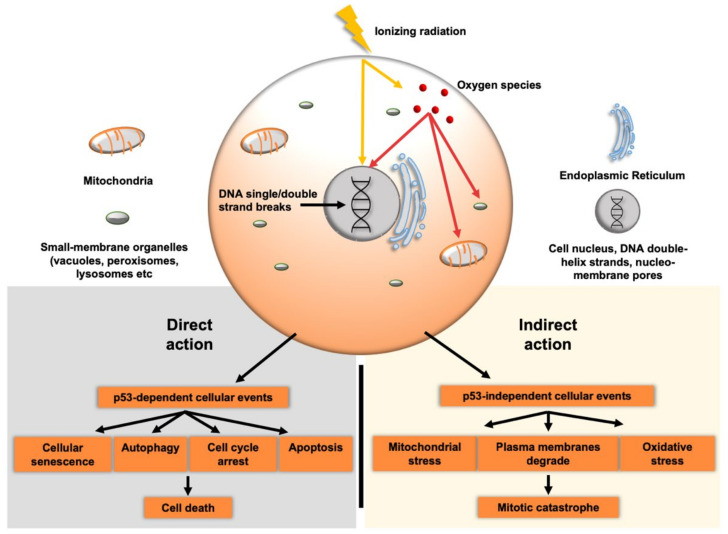
Cellular effects of ionizing radiation. Ionizing radiation directly damages DNA within targeted cells, causing both single and double strand DNA strand damage. Oxygen species are also created that cause DNA damage and disrupt lipid membrane components of the cell. Both the direct and indirect actions of radiation therapy cause downstream effects that lead to mitotic catastrophe and cell death.

In concert with these repair processes, a range of other cellular pathways are activated in response to RT that interface with DNA repair. A predominant molecular feature of this DNA damage response involves the activation of p53, which functions to mediate apoptosis, cell cycle arrest or induction of cellular senescence. This is visualized within Figure 1 which highlights both direct and indirect action of RT. p53 maintains a pivotal role in execution of these signal pathways, while the cellular outcome is dependent upon the threshold of DNA damage and levels of p53 protein [13].

### 2.2. Indirect Effects

Reactive oxygen species (ROS) are the predominant effector of the indirect damage that RT induces when applied to malignant tissue. Despite initial inclinations, ROS are an important driver of cellular homeostasis, intracellular and cell-cell signaling and a normal response to microbial invasions [14]. During normal cellular homeostasis, the ROS system is tightly regulated by the intracellular antioxidant system which neutralizes these potentially harmful molecules [15]. However, when this antioxidant system is overwhelmed, either due to cytotoxic conditions, necrosis, mitochondrial damage or other stresses, then cellular signs of oxidative stress begins to present [16].

The indirect mechanism of action of RT in treated tissue and the respective cell populations present is through the radiolysis of water, which generates intracellular ROS, causing mass cell damage [17,18]. Further, the ionizing radiation of RT can also affect the mitochondria, thereby, creating a secondary source of intracellular ROS generation and further overwhelming the antioxidant defense system [19,20].

RT causes cellular damage in several ways and modulates apoptosis signaling. DNA damage, mitochondrial stress, and lipid-membrane degradation are typical sites of disfunction due to ROS, though interestingly, p53 has been explored and found to mediate some of these effects. [21]. One such indirect impact of RT is the oxidation of DNA bases generated by ROS. Removal and repair of DNA lesions requires the base excision repair (BER) pathway. This pathway is catalyzed by sequential damaged base recognition and removal enzymes (glycosylases and an AP-endonuclease), which generate a SSB, followed by a DNA polymerase and ligase to replace the base and reconstitute the DNA [22]. Additionally, the upregulation of ROS within a cell oxidizes the plasma bilipid membrane, called lipid peroxidation. This leads to enhanced membrane permeability, resulting in the disruption of trans-membrane and membrane-bound proteins. This ultimately affects the homeostatic function of the plasma membrane [23]. 

An important player in the response to ROS is p53. Perillo et al. comment on the debated role of p53 in upregulated ROS cells, stating that p53 can both promote oxidant and antioxidant gene expression [24]. Furthermore, p53 has been involved in the upregulation of a damage-regulated autophagy modulator that results in cytoprotective behavior through organelle recycling due to ROS damage [21]. These studies imply that though RT can cause system intracellular damage through upregulated ROS, cancer cells can respond through many signaling and effector mechanisms to mediate ROS effects, and in some cases, utilize ROS to their advantage, potentially indicating mechanisms of action of radio-resistance.

## 3. Clinical Issues of Radiotherapy and Associated Phenomena

The clinical treatment of breast cancer with ionizing radiation is quite common and uses different techniques to induce cancer cell death. This is dependent upon staging and the progression of the cancer. For example, partial breast irradiation focuses on the tumor tissue bed and aims to minimize exposure to surround tissue [25]. Despite best efforts by clinicians, it is near impossible to preserve normal tissue with current modalities and technologies. Clinical side-effects arise during RT treatment that ultimately lead to treatment changes, stopping of treatment, secondary cancers, and radiation resistance. One significant concern is cardiotoxicity or cardiopulmonary toxicity. Cardiomyocytes are typically resistant to radiation due to their stability and lack of proliferative potential, though, it has been reported that with modern RT techniques, they can sustain damage which can be seen within the microvasculature of the myocardium [26,27]. Additionally, pericardial inflammation can occur within breast cancer patients treated with RT [28,29]. At a more local level, the bystander effect can arise. This effect describes local normal tissue and cells near to the irradiation site that sustain cellular damage due to extracellular ROS molecules. Novel RT modalities will need to minimize toxicity in both neighboring tissue, such as what has been observed with cardiomyocytes, and local normal cells that suffer from the indirect cytotoxicity effects of RT [30].

Radio-resistance is another growing concern among clinicians and there is a need to not only better understand this obstacle but also avoid the development of resistance. It has been reported previously that changes in the mechanistic target of rapamycin (mTOR), phosphatidylinositol 3-kinase (PI3K), Ras-mitogen-activated protein kinase (MAPK) and signal transducer and activator of transcription (STAT) are upregulated and serve signal pro-survival behaviors [31]. Both the cellular and non-cellular components of the tumor microenvironment (TME) contribute and assist with pro-survival behavior of the cancer. Like all treatments, resistance to RT is a concern of clinicians and proves to be a significant obstacle in the successful treatment of not just breast cancer but by all cancers treated with RT.

Additionally, there is a growing body of evidence that demonstrates the increased risks of late secondary malignancies forming due to low levels of non-targeted irradiation in patients who receive RT. When treating primary breast cancer with RT either locally or regionally, the relative risk (RR) of secondary lung cancer developing within 5-years increased by 1.39 (95% CI, 1.28–1.51). By the 15-year mark, RR increased to 1.66 (95% CI, 1.01–2.01 [32,33]. Similarly, it was reported that genetic susceptibility, lifestyle, environmental factors and other treatments such as chemotherapy further increased specific cancer risks and when combined with RT, further increased second cancer occurrences [34,35,36].

Further, there are additional detrimental effects of RT upon cancers that can lead to upregulation of metastatic behaviors, such as immunosuppression and local tissue damage [36]. Irradiated normal tissue can transform into a hospitable microenvironment and have a higher propensity to be sites of metastasis [37,38]. RT induced metastasis and secondary cancers are significant hurdles that need to be addressed, either through further research or by the determination of a multi-modal treatment system that can counter metastatic behavior or maintain immune-competence within patients.

## 4. Current Preclinical Tools to Evaluate Effects of Radiation Therapy

In spite of the abovementioned issues, we have continued to explore RT for its beneficial effects in both clinical and research settings. There are currently 136 ongoing clinical trials in the world that use RT alone or in combination with other modalities as an intervention for the treatment of breast cancers (Source: https://clinicaltrials.gov/, accessed on 30 September 2021). Some of the trials are aimed at optimizing the dosage regimen and testing effectiveness of different delivery techniques of RT. And others are aimed at understanding the effectiveness of combinations of chemotherapy or hormonal therapy with RT. There exists a huge disparity in the number of reports on preclinical radiation research and the numbers that eventually reach clinical trials. To drive effective large-scale, expensive and time-consuming early phase clinical trials, we need promising data from pre-clinical studies. To increase the translatability of results from these pre-clinical studies, reproducible and relevant in vitro and in vivo models should be developed to investigate radiation effects and response [39]. Across the wide range of in vitro models, there are several techniques of radiation application, different dose regimens, different types of cells (primary and immortalized, human-derived and animal-derived) and distinct geometries [40]. Consistent approaches, standardized parameters and specific end-point analyses need to be established in pre-clinical models to determine the effectiveness of RT. Pre-clinical studies are crucial to understand radiation biology and study mechanisms of radiation resistance. Such studies have been very useful to elucidate the critical role of hypoxia in regulating the resistance of tumor cells to radiation [41,42,43]. Pre-clinical models are also needed for the investigation of interventions to overcome radiation resistance, including the use of radiosensitizers, which are agents that can increase the sensitivity of the cells or tissue to irradiation [44].

### 4.1. Animal Models

While RT is a localized treatment modality, the responses are exhibited at a systemic level and studies have tried to exploit these responses using pre-clinical animal models [45]. Predominantly, in vivo studies have evaluated methods to overcome radiation resistance of breast cancers. Delayed tumor growth was observed in a Triple Negative Breast Cancer (TNBC) mouse model when a drug identified using in vitro studies, Mebendazole, was used in combination with RT [46]. Atkinson et al. utilized gold nanoshells in combination with infrared radiation to enhance radiation sensitivity of cancer stem cells in xenograft model of TNBC [47]. In another study, targeted bismuth nanoparticles were assessed for increasing the sensitivity of X-ray radiation therapy of breast cancer [48]. Radiation can also cause tumor recurrence by recruitment of circulating tumor cells. Using an orthotopic model of breast cancer, Vilalta et al. demonstrated that this was mediated by radiation-induced granulocyte-macrophage colony-stimulating factor (GM-CSF) production by tumor cells [37]. Exploring the role of stroma in radiation resistance, Steers et al. demonstrated that the addition of fibroblast in a breast cancer xenograft model increased tumor progression and induced radiation resistance [49]. Investigation of combined effects of RT and immunotherapy [50], and the study of abscopal effects of irradiation [45] require a functional whole-body response making animal models a necessity to study such effects.

Further, animal models provide valuable platforms to study radiation related systemic toxicity effects. Focused reviews have commented on the utility of animal models to study radiation related cardiac toxicity [51], pulmonary toxicity [52] and such multi-organ toxicity studies will need the sophistication of an in vivo model. However, we need to recognize the innate drawback of poor translation of non-human, xenograft responses into patient outcomes. Also, the differences between radiation set ups and parameters for animal studies when compared to clinics need to be considered while interpreting these results [53]. While animal models will remain crucial in testing systemic effects prior to clinical studies, effective in vitro 2D and 3D models can considerably reduce the load on pre-clinical animal testing. Additionally, humanization of 3D models allows the investigation of personalized RT, which may enable the patient-specific prediction of radiation response more so than animal models.

### 4.2. 2D Models

As a vital therapeutic modality used in the clinical intervention of breast cancers, RT has been studied extensively in 2D cultures using breast cancer cell lines such as T47D, MCF-7, MDA-MB-231, MDA-MB-468 and MDA-MB-361. Most of the studies have used doses ranging from 0 Gy to 8 Gy using different irradiation sources and varying dose rates. A strong focal point of these 2D studies has been the investigation of ways to enhance radiosensitivity of the cells to overcome radiation resistance. One of the commonly studied methods is the use of metallic nanoparticles [54,55,56] and magnetic nanoparticles [57,58] to improve the therapeutic efficiency of radiation treatment of cancer cells. Irradiated nanoparticles enhance the dose deposited locally by producing secondary electrons that add to ROS production and DNA damage within the cell [56]. Another important strategy has been to repurpose already known small molecule inhibitors and chemotherapeutic drugs to complement irradiation and function as radiosensitizers [59,60,61,62]. In a recent study, Speers et al. analyzed the radiation responses of 21 breast cancer cell lines using a high-throughput novel drug radiosensitivity screen where the androgen receptor was identified as the optimal target for radiosensitization [63]. Histone Deacetylase (HDAc) inhibitors are another frequently studied group of inhibitors that have a selective toxicity towards cancer cells [64]. By inhibiting the activity of HDAc, these inhibitors cause a decondensation of the chromatin structure [65] and reduce the ability of cells to repair DNA damage [64]. Consequently, these HDAc inhibitors have been used in radiosensitization studies to study their effects on cancer cell responses [65,66,67]. Radiosensitivity observed in solid tumors such as breast cancers has also been attributed to the presence of hypoxia [42]. Targeting this hypoxic core, studies have shown that breast cancer cells elicit better radiation responses by the inhibition of antioxidant enzymes [68,69] or targeting tumor metabolism [70] as a result of increased oxidative stress. While there is a lot of focus on finding potential radiosensitizers, studies have also tried to identify radioprotectors that are equally important to reduce the toxicity of radiation in normal tissues [71,72]. Normal tissues or non-targeted cells may also be affected via soluble factors that are secreted by the tumor cells in response to radiation by what is known as the bystander effect. This effect warrants research because of its role in tumor recurrence and metastasis. A couple of 2D studies have profiled the soluble factors including cytokines, receptors, exosomes, etc., secreted by cancer cell lines in the conditioned medium and evaluated the effects of the medium on the growth of bystander tumor cells or endothelial cells [73,74,75]. Jabbari et al. had demonstrated that the increased radiation doses resulted in an increase in the secretion of vascular endothelial growth factor (VEGF-A) from MCF7 cells [74]. The conditioned medium from irradiated MCF7s was shown to enhance the angiogenic responses in endothelial cells that may potentially aid with generation of secondary tumors [74].

Furthermore, radiation responses have been predicted to be associated with the specific genomic and proteomic traits of the breast cancer cells [76,77]. Clinically, luminal sub-types like ER, PR-positive breast cancers seem to have better responses to RT when compared to the basal-subtypes including HER2 positive breast cancers and TNBCs. However, the dependency of radiation response on the subtype of breast cancer is still unclear [78,79]. Bravata et al. demonstrated that IR-induced gene expression profiles and pathways appear to be cell-line dependent [79]. On the other hand, Speers et al. did not find a correlation between radiosensitivity and breast cancer sub-type [76]. This is further complicated by the intratumoral and intertumoral heterogeneity of breast cancers [80]. Gao and colleagues have shown the heterogeneity of cellular response to irradiation using single cell sequencing of breast cancer cells [81]. These data signify the need for a personalized approach in the breast cancer treatment using adjuvant RT [82].

While 2D studies have enabled better understanding of radiation responses of breast cancer cells, monolayer cultures are oversimplified and lack the complex 3D architecture of the TME. This would mean that they cannot recapitulate the intercellular, intracellular and cell-ECM interactions that determine the tumor growth, metastasis and response to targeted therapy [83]. Inadequacy of these models diminishes the potential of translating the findings to clinical settings. With regards to RT, the modality relies heavily on its ability to cause DNA damage to the cells which eventually results in cell death. However, it has been shown in several studies that this effect is altered in the presence of an ECM that confers radioresistance to the cells. Cells cultured on ECM substrates (2.5D cultures) have demonstrated an increase in surviving fractions post irradiation [84,85]. This resistance to radiation has been contributed to a phenomenon called cell adhesion-mediated radioresistance (CAM-RR) [86,87]. Substrate coatings of ECM proteins including fibronectin and laminin have been shown to cause a reduction in the sensitivity of the breast cancer cells to ionizing radiation [85]. This reduced sensitivity has been attributed to CAM-RR which in turn has been shown to be a consequence of strong integrin clustering at the site of cellular adhesion to the matrix, aiding in better cell survival when compared to 2D cells [88]. Studies have unraveled molecular mechanisms and signaling pathways that enable the integrin-mediated radioresistance in 3D cultures [89,90,91]. Consequently, there is a shift towards using 3D models to study radiation biology and identify radiosensitizers.

### 4.3. 3D Models

In 3D radiobiology studies, researchers have worked with scaffolds, hydrogels, and spheroids to incorporate the third dimension for a wide range of cancers including breast cancers [92], brain tumors [93,94], lung cancers [95,96], bladder cancer [97], chondrosarcoma [98] and oral squamous cell carcinoma [99,100]. The majority of these studies have demonstrated the differential effects of RT on 2D vs 3D cultures.

Table 1 presents a list of in vitro 3D breast cancer models that have been treated with a range of radiation doses, dose rates, and set ups and reported the effects of irradiation on the cells in 3D. Over half of the radiation studies have cultured 3D breast cancer tissues in the form of spheroids generated using a scaffold-free, low attachment plate technique [101]. As the name suggests, these cultures do not possess external matrix components and are formed by self-assembly governed by cell-cell interactions. Remaining studies have used reconstituted basement membrane-based substrates such as Matrigel where the cell-substrate interaction dominates the 3D cell attachment. Expectedly, 3D cultures show varying radiation responses when compared to the monolayer cultures [102,103]. With an upregulation of expression of stem cell-like markers, the 3D cultures have been shown to present increased resistance to radiation [103,104].

Like the 2D studies discussed in the previous section, the 3D studies have also been targeted towards identification and evaluation of radiosensitizers. Complementing radiation treatment with drugs including Olaparib [105], Valproic acid [106], Mebendazole [46], Vinblastine [107], Trastuzumab [108], Vorinostat [109], Simvastatin [92], has been shown to affect the radiosensitivity of breast cancer cells. Interestingly, some of these studies have shown varying responses to the same drug in the monolayer culture vs 3D cultures. Valproic acid, a HDAc inhibitor, had a radioprotective effect in 3D mammosphere cultures of breast cancer cells, whereas it had a radiosensitizing role in monolayer cultures [106]. This difference in observed effects of Valproic acid has been attributed to the self-renewal promoting culture conditions in 3D when compared to the differentiated cell cultures of 2D [106]. In another study, Simvastatin could effectively radiosensitize monolayer cultures of normal and cancerous breast cells but did not elicit a radiation response in 3D cultures [92]. Again, these results highlight the importance of working with physiologically relevant 3D models during the preclinical phase.

Further, some of the 3D studies have incorporated co-cultures with CAFs [49,107,110] and endothelial cells [110,111] to recapitulate the complexity of the tumor environment and understand its effects on radiation responses. Upreti et al. showed that the co-cultures of tumor cells with endothelial cells in 3D increased the sensitivity of the cultures to chemotherapy and simultaneously, protected the tumor cells from irradiation [111]. Co-cultures of cancer cells with fibroblasts have shown contradicting effects in radiosensitivity studies. In a spheroid-based study that assessed optimal combinations of chemotherapeutic drugs and radiation, results showed that culturing fibroblast with cancer cells did not alter radiation sensitivity [107]. On the other hand, co-cultures of cancer cells with fibroblasts in Matrigel enhanced cell survival post-irradiation, thereby demonstrating the radioprotective nature of these stromal cells [49]. This may be explained by the presence of cell-ECM interactions in Matrigel-based cultures conferring the cultures with enhanced radiation resistance. While several studies have evaluated the role of stromal cells in mediating chemotherapeutic resistance [112,113], fewer studies have been conducted to elucidate their potential in regulating radiation resistance [87,114], especially in 3D cultures. This clearly shows the need for further investigations into studying the responses of stromal cell co-cultures with breast cancers cells in 3D radiobiology studies.

While there have been continued efforts to enhance radiation responses of cells in 3D breast cancer cultures, limited research has been conducted to elucidate the underlying mechanisms behind these effects. Mechanistic studies have been conducted using monolayer cultures [115] with only a few 3D studies that have evaluated the effects using skin and airway tissues [116,117]. There is an unmet need to unravel the molecular mechanisms behind the radiation responses in 3D cancer models. Nevertheless, few mechanistic studies that have been undertaken to understand the cell-adhesion mediated radiation resistance observed in the 3D ECM-based breast cancer cultures. Studies using 3D laminin-rich ECM (lrECM) cultures have found that a β1 integrin-dependent signaling pathway aids in the survival of cells post irradiation [89,90,118]. Particularly, Nam et al. showed that inhibition of a specific integrin heterodimer (α5β1-integrin) increased the efficacy of irradiation and caused breast cancer cell apoptosis in lrECM cultures [90]. Additionally, Ahmed et al. identified the role of NF-kB in mediating this radiation-induced upregulation of b1-integrin expression in malignant breast cancer cells, which eventually resulted in radiation resistance and pro-survival signaling [89]. Further studies will be necessary to assess the possibilities of establishing direct and indirect means of DNA in 3D models, evaluating adjacent normal tissue damage and characterize DNA damage pathways in 3D.

An important consideration in 3D studies which is often overlooked is the recapitulation of the dynamic nature of the tumor microenvironment. This becomes quite relevant because of its role in resistance to therapy. It has been shown that interstitial fluid pressure (IFP) is enhanced in most solid tumors, including breast carcinomas [119,120]. And interestingly, studies have correlated this increase in IFP with disease progression and poor prognosis after receiving radiation therapy [121,122]. Within the tumor microenvironment, the enhanced IFP results in the exposure of cancer cells to enhanced interstitial fluid flow.

Several studies have mimicked this interstitial fluid flow by in vitro application of shear stresses on breast cancer cells. This has been shown to have an effect on cellular proliferation [123], stemness [124], motility [125], and chemoresistance [126]. Clearly, it becomes crucial to incorporate and evaluate these shear stresses in 3D breast cancer models as well. Additionally, fluid flow also becomes quite relevant in 3D studies because it can aid in nutrient diffusion and in the maintenance of cellular viability for long term studies of large-volume tissues [127]. Studies have utilized perfusion bioreactor set ups to evaluate the effects of fluid flow in 3D breast cancer tissue models. In a study by Shields et al., physiologically relevant fluid flow was shown to cause breast cancer cell chemotaxis to the lymphatic system using a 3D Matrigel-based breast cancer model in co-culture with lymphatic endothelial cells [128]. Here, they demonstrate a potential tumor cell metastasis pathway induced by interstitial fluid flow. In another study, Novak et al. applied pulsatile fluid flow on breast cancer cells embedded in 3D hydrogels using a 3D shear stress bioreactor [129]. They observed an upregulation of cellular proliferation and altered cell morphology in the presence of a shear stress stimulus. More importantly, the breast cancer cell lines showed resistance to chemotherapy (Paclitaxel) with more surviving cells upon shear stimulation [129]. In a similar study, Azimi et al. demonstrated a reduction in 3D breast cancer sensitivity to Doxorubicin in the presence of a fluid flow environment [130]. With these studies indicating the role of fluid flow on breast cancer metastasis and resistance to chemotherapy, its effects on radiation response remains unanswered. Using a microfluidic platform with MDA-MB 231 cells encapsulated in collagen gels, Polacheck et al. demonstrated a β1 integrin mediated focal adhesion reorganization induced by interstitial fluid flow [131]. This is closely related to the signaling pathways in 3D radiation resistance mechanisms described previously. In a 2D study with colon cancer cells, shear stress was shown to enhance radiosensitivity of tumor cells via β1 integrin/ focal adhesion kinase signaling pathway [132]. While these may indicate the potential role of shear stresses in modulating radiosensitivity, there is lack of research on the influence of dynamic fluid flow in breast cancer radiobiology. With advancements in 3D dynamic cultures systems and increased appreciation of its relevance in therapeutic responses, there is a definite need to evaluate these effects in future radiobiology studies.

**Figure 2:**
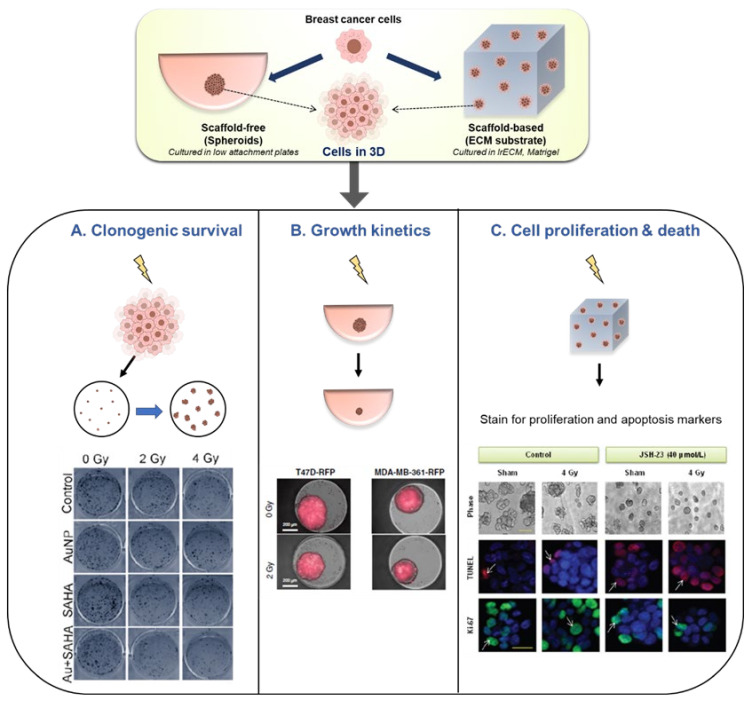
Analysis methods in radiobiology models of 3D breast cancer tissues. Spheroid (scaffold-free)-based and substrate-based cultures have been used to study in vitro radiation response in breast cancer cells. Some representative studies along with their methods of analysis of radiation effects are presented here. (**A**) Clonogenic survival assay: Cells are irradiated in 3D, then they are isolated and replated in 2D at low densities to assess their colony formation abilities. Evaluation of colony forming ability of irradiated MCF7 spheroids (reproduced from [109]). (**B**) Growth Kinetics: Spheroid cultures are irradiated and the effect on cell growth is identified by measuring changes in spheroid size. Evaluation of growth of breast cancer spheroids by measuring tissue area using constitutive lentiviral-RFP expression (reproduced from [107]). (**C**) Cell proliferation and death: Evaluation of radiation induced cell death and decrease in proliferation in T4-2 cells in laminin rich ECM cultures (reproduced from [90]).

In the studies presented in Table 1, we observe that some of the common methods of analysis include the evaluation of clonogenic survival, tissue growth, cell proliferation and death, and metabolic activity (Figure 2). Clonogenic survival assay is a gold-standard, well-established test used in radiobiology experiments to determine the cell’s ability to form a clone upon irradiation. It is a time-consuming assay and more commonly used with monolayer cultures. Most of the studies in Table 1 have relied on disrupting the 3D structure of both the spheroid-based models [109,111] and substrate-based models [103] to re-plate the cells in 2D for the clonogenic survival assay. For instance, Igaz et al. resuspended cells from MCF7 spheroids post irradiation, reseeded them in 2D followed by a week of culture before the counting the colonies (Figure 2A) [109]. The results showed the reduced potential of irradiated MCF7 spheroids to form colonies. There is still a need to find alternative techniques for analyzing the this assay as the purpose of using 3D cultures is lost to some extent when the cells need to be re-plated and cultured in 2D. Future studies could explore real-time or automated, image-based clonogenic survival analyses of 3D tissues presented in the literature [49,133,134].

Another useful method of analysis is the non-destructive assessment of tissue growth kinetics in scaffold-free, spheroid-based models [107]. Anastasov et al. evaluated 3D microtissue growth by calculating tissue area in breast cancer spheroids of T47D and MDA-MB-361 in response to different radiation doses (2–8 Gy) [107,108] (Figure 2B). This enabled the comparison of radiation sensitivity of the two cell types and the method was also used for studying the radiosensitizing effects of established chemotherapeutic drugs [107]. As one would expect, radiation has been predominantly studied with an aim for causing cellular death or cell cycle arrest. Consequently, markers for cellular proliferation, cell death and DNA damage have been widely studied in radiation experiments. Ahmed et al. had used TUNEL staining and Ki-67 staining to detect apoptosis and proliferation respectively in irradiated T4-2 cells in 3D lrECM cultures [89] (Figure 2C). γH2AX a well-known marker of DNA double stranded breaks which is induced by irradiation. It has been used for assessing DNA damage post irradiation in several radiation biology studies [135]. Yet, it has not been explored as widely in in vitro 3D breast cancer radiobiology studies. In addition to the assessment of growth and proliferation, studying radiation induced changes in gene and protein expression has helped to understand the cellular mechanisms of radiation response. A radiation dose of 2 Gy was shown to cause an upregulation of surface expression of integrin in 3D lrECM cultures of malignant breast cancer cells, which was measured by both immunoblotting and immunofluorescent staining [90].

Despite the above-mentioned examples, the lack of consistent methods of analyses in 3D radiobiology studies is a limiting factor in the translation of radiation responses into preclinical and clinical investigations. There exists ample scope to develop novel analysis techniques and a need to establish better markers for assessing the effectiveness of radiation treatments in 3D models.

## 5. Future Directions in 3D Breast Cancer Radiobiology Models

The last decade has seen a surge in the number of in vitro radiobiology studies that have used 3D models of cancer tissues. Specifically, research on testing drug-radiation combinations using 3D breast cancer models has been a major focus of interest. However, studies conducted so far have used scaffold-free spheroids or Matrigel for their 3D tissue culture. While scaffold-free tumor spheroid generation is a relatively simple and reproducible technique, it relies on matrix deposition by the cells, a crucial factor involved in radiation resistance associated with cell-ECM interactions. While Matrigel has the ability to replicate the cell-ECM interactions, it is associated with high batch-to-batch variability [138] and uncontrolled degradation which could potentially cause variable outcomes. It may be more beneficial to use tunable, reproducible biomaterials that mimic the native ECM of the breast tissue in 3D radiobiology studies. Extensive research on the use of scaffolds and synthetic hydrogels for developing breast cancer models [139,140,141], especially in chemotherapy studies, offers easier access for repurposing these models for radiobiology studies. However, it may still be a challenge to work with the inconsistent effects observed across the different cell/biomaterial/radiation combinations. Additionally, RT experiments have another variable factor in the form of different radiation systems. Efforts to standardize in vitro dosimetry studies may be of significant help to address these issues [142]. Another important facet of physiological systems is the dynamic nature of native microenvironment. A few radiobiology studies have explored this aspect using tumor-on-chip devices that can provide controlled fluid flow to the irradiated 3D cultures [143,144,145,146]. The potential of these microfluidic systems for exploring breast cancer radiation responses remains largely unexplored.

The next challenge would be to recapitulate the complexity of the heterogeneous nature of the breast cancer tissue. Going forward, a stronger shift from monocultures to co-cultures can be expected in 3D cultures, given its relevance in radiation resistance. Another important aspect of consideration is the personalization of RT using patient-specific cells. Considering the variable patient-dependent responses observed in clinical RT, there is a clear need to move beyond immortalized breast cancer cell lines in 3D studies. Further, there is active research happening in the field of genomic-driven personalization of RT [147,148]. Biological signatures identified by sequencing mRNA, whole genome, miRNA, single nucleotide polymorphisms analyses are being studied to identify prognostic markers to predict radiation response [149,150]. Recently, Aristei et al. summarized clinical trials that have evaluated patient radiosensitivity using genomics analyses [151]. Based on genetic profiling, patients can be stratified for potential beneficial effects of adjuvant RT. Similarly, normal tissue radiotoxicity effects can also be potentially linked to specific genomic signatures in patients [152]. With rapid advancements in 3D tissue engineering, there is a huge potential for advancing preclinical radiation research in breast cancers to study personalized effects and drive translation of the outcomes to effective clinical trials. Continued efforts to develop and standardize 3D radiation biology protocols can accelerate the search for effective radiosensitizers and contribute to identifying predictive biomarkers of personalized radiation response.

## Figures and Tables

**Table 1:** In vitro radiobiology studies conducted using 3D models (spheroid-based and substrate-based) of breast cancer tissues.

S No	Cells Matrix (Substrate-Based)	Radiation Dosage	Dose Rate	Observations (Radiation Effects)	Methods of Analysis	Ref.
		Irradiator				
**Spheroid-Based Models**						
1	MDA-MB-231, SUM1315	2 Gy x 5	400 (MU/min)	**Radiosensitizer**: Olaparib in low doses with longer exposure team enhances radiosensitivity	Metabolic activity, live/dead analysis	[105]
		Varian Medical Systems				
2	MCF-7, primary human breast cancer	2, 4, 6 Gy		**Radiosensitizer:** Valproic acid radiosensitizes in 2D and radioprotects in 3D	GelCount Colony Counter	[106]
		Cs-137 irradiator				
3	GFP-4T1 + 2H11 murine endothelial cells	2 Gy		**Co-culture:** Presence of endothelial cells sensitized cells to chemotherapy and protected tumor cells from irradiation	2D replating and survival assay	[111]
		Not stated				
4	SUM159PT, MDA-MB-231	4, 8 Gy	2.789 Gy/min	**Radiosensitizer:** Mebendazole inhibited IR-induced conversion of TNBC cells into cancer-initiating phenotype	Mammospheres count	[46]
		X-ray irradiator Gulmay Medical Inc				
5	T47D, HTB-133, MDA-MB-361, MDA-MB-231 + primary normal human dermal fibroblasts	2, 4, 6, 8 Gy	0.5 Gy/min	**Radiosensitizer and co-culture:** Vinblastine and radiation inhibited cancer cell growth	Image-based analysis of tissue area	[107]
		Cs-137 irradiator				
6	T47D, JIMT-1	5 Gy	0.95 Gy/minute	**Radiosensitizer:** Trastuzumab and radiation inhibited growth in 3D	Image-based analysis of tissue area	[108]
		Cs-137 irradiator				
7	4T1-mCherry tumor cells, C166-GFP endothelial cells, murine embryonic fibroblasts	3 Gy	1.018 ± 0.10 Gy/min	Radiation enhances expression of Galectin-1 in endothelial cells that is targeted using nanoparticles carrying arsenic trioxide and cisplatin	Dead cell staining (Sytox blue)	[110]
		Varian TrueBeam System				
8	MCF-7	2, 4, 6, 8 Gy	200 MU/min	Increased radiosensitivity in 3D compared to 2D Curcumin enhances radiosensitivity of cancer cells	MTT assay, RT-PCR, ELISA	[102]
		PRIMUSTM linear accelerator				
9	A549 lung adenocarcinoma, DU-145 and PC-3 prostate cancer and MCF-7 breast cancer	0, 2, 4 Gy		**Radiosensitizer:** Gold nanoparticle, Vorinostat and radiation reduces colony forming ability of cells and enhanced DNA damage	2D replating and clonogenic survival assay, γH2AX staining	[109]
		Primus linear accelerator				
**Substrate-based Models**						
10	MCF7, MDA-MB-231, SK-BR-3 Matrigel	2, 6 Gy		Gene expression of CSC depended on radiation dose Radiation had differing effects on expression of MMP, TIMP and HDAc	RT-PCR	[136]
		Yxlon Smart Maxishot 200-E				
11	T4-2 (malignant) GFR BME (laminin-rich ECM)	2, 4 Gy		IR caused upregulation of integrin leading to increased cell survival Inhibition of integrin induced apoptosis	Immunoblotting, immunofluorescence, TUNEL assay	[90]
		Not stated				
12	184A1 human MamECs Matrigel	0.5, 1, 2.5, 5 Gy	0.16–0.58 Gy/min	Reduced apoptosis in 3D compared to 2D Increased survival in long-term 3D cultures because of growth inhibition in 3D	Trypsinize and count, flow cytometry	[104]
		Pantak XRAD 320 Cabinet X-ray machine				
13	MCF10a, 184v human MamECs Matrigel	0.4–2 Gy	X-ray: 4 Gy/min, 0.03 Gy/min γ: 0.03–2 Gy/min, ^56^Fe: 0.2–1 Gy/min	E-cadherin was reduced in TGF-β–treated cells irradiated with radiation TGF-β–mediated EMT is not dependent on radiation dose or quality	Immunofluorescence (cryosections)	[137]
		X-rays: Varian 2300 linear accelerator				
14	T4-2 (malignant), S1 (non-malignant) GFR BME (laminin-rich ECM)	0–8 Gy		Integrin induced by exposure to radiation through NFkB–mediated gene activation in 3D	Western blot, RT-PCR, NF-kB DNA-binding assay, immunofluorescence, TUNEL assay	[89]
		Not stated				
15	A549 adenocarcinoma, MCF7, PC3 prostate cancer Matrigel	0–4 Gy	0.751 Gy/min	3D cultures have increased radioresistance	2D replating and survival assay	[103]
		Faxitron RX-650 facility				
16	Py8119, NIH-3T3 Matrigel	3, 6, 9 Gy	3 Gy/min	**Co-culture:** Presence of fibroblasts increased the survival fraction of irradiated cultures	Survival assay and fluorescence	[49]
		Isovolt-320-X-ray machine				
17	MCF10A, MCF7 Matrigel	0.5, 2, 4, 6 Gy	3.75 Gy/min	**Radiosensitizer:** Simvastatin tends to radiosensitize in 2D and not in 3D	3D clonogenic survival assay	[92]
		Linac Siemens Oncor Expression				
		Not stated				

MU/min: Monitor Units per minute; Cs-137: Caesium-137; GFP: Green Fluorescent Protein; IR: Irradiation; TNBC: Triple-negative breast cancer; MTT: 3-[4,5-dimethylthiazole-2-yl]-2,5-diphenyltetrazolium bromide; RT-PCR: Real Time polymerase chain reaction; ELISA: Enzyme-Linked Immunosorbent Assay; γH2AX: gamma Histone-2AX; CSC: Cancer Stem Cells; MMP: Matrix metalloproteinases; TIMP: The tissue inhibitors of metalloproteinases; HDAc: Histone deacetylase; GFR: Growth Factor Reduced; BME: basement membrane extract; ECM: Extracellular Matrix; TUNEL: Terminal deoxynucleotidyl transferase (TdT) dUTP Nick-End Labeling; MamECs: mammary epithelial cells; TGF-β: Transforming growth factor beta; EMT: Epithelial to Mesenchymal transition; NFkB: Nuclear Factor kappa-light-chain-enhancer of activated B cells; DNA: Deoxyribonucleic acid.
